# Investigating seizures in bariatric surgery patients: Pre- and postoperative perspectives: A systematic review

**DOI:** 10.1016/j.obpill.2026.100326

**Published:** 2026-09-02

**Authors:** Matin Bidares, Mahsa Aziz, Sara Samadzadeh, Ramtin Bidares, Sjaak Pouwels, Suhaib J.S. Ahmad, Carolina Pape-Köhler

**Affiliations:** a Health Policy Research Center, Institute of Health, Shiraz University of Medical Sciences, Shiraz, Iran; b Experimental and Clinical Research Center, Charité – Universitätsmedizin Berlin, Core member of Freie Universität Berlin and Humboldt-Universität zu Berlin, Berlin, Germany; c Department of Clinical and Molecular Medicine, Sapienza University, Rome, Italy; d Department of Surgery, Bielefeld University Medical School and University Hospital OWL – Campus Klinikum Lippe, Detmold, NRW, Germany; e Department of Surgical Oncology, Transplant Surgery and General Surgery, Medical University Gdansk, Gdansk, Poland; f Health Education and Improvement Wales, Wales, UK; g Bariatric Clinic, Klinikum Bielefeld – Rosenhöhe, Bielefeld, Germany; h Bielefeld University, Medical School and University Medical Center OWL, Klinikum Bielefeld-Mitte, Department of General and Visceral Surgery, Bielefeld, Germany

**Keywords:** Seizures, Bariatric surgery, Antiepileptic drug, Neurological complications

## Abstract

**Background:**

Bariatric surgery is widely used for severe obesity and may influence neurological outcomes through metabolic alterations, nutritional deficiencies, and changes in Antiepileptic Drug (AED) absorption. Despite increasing reports of neurological complications, seizure patterns before and after bariatric surgery have not been comprehensively summarized.

**Methods:**

This was a systematic review conducted according to Preferred Reporting Items for Systematic Reviews and Meta-Analyses (PRISMA) guidelines. A systematic search of PubMed, Scopus, Web of Science, and Embase was conducted through April 20, 2025. Eighty-three studies reporting seizures before and/or after bariatric surgery were included. Data on patient demographics, surgery type, seizure timing, etiology, AED adjustments, and clinical outcomes were narratively synthesized.

**Results:**

Across the included studies, patients were mostly middle-aged women undergoing sleeve gastrectomy or Roux-en-Y gastric bypass. Three outcome patterns emerged. Some patients with pre-existing epilepsy remained seizure-free postoperatively with stable AED regimens. Others experienced persistent or recurrent seizures, commonly associated with reduced postoperative absorption of valproate or phenytoin, requiring dose escalation or drug substitution. New-onset postoperative seizures occurred across both surgery types and were predominantly secondary to metabolic disturbances especially hypoglycemia, hyperammonemia, electrolyte abnormalities, or cardiac arrhythmia and typically resolved after addressing the underlying cause. AED adjustments were more frequently required after gastric bypass due to greater pharmacokinetic changes.

**Conclusion:**

Seizure outcomes after bariatric surgery are variable and influenced by altered drug absorption and postoperative metabolic complications. Most patients maintain seizure control, but a subset develops breakthrough or new-onset seizures, highlighting the need for AED monitoring and careful evaluation of metabolic causes.

## Graphical abstract

**Figure:**
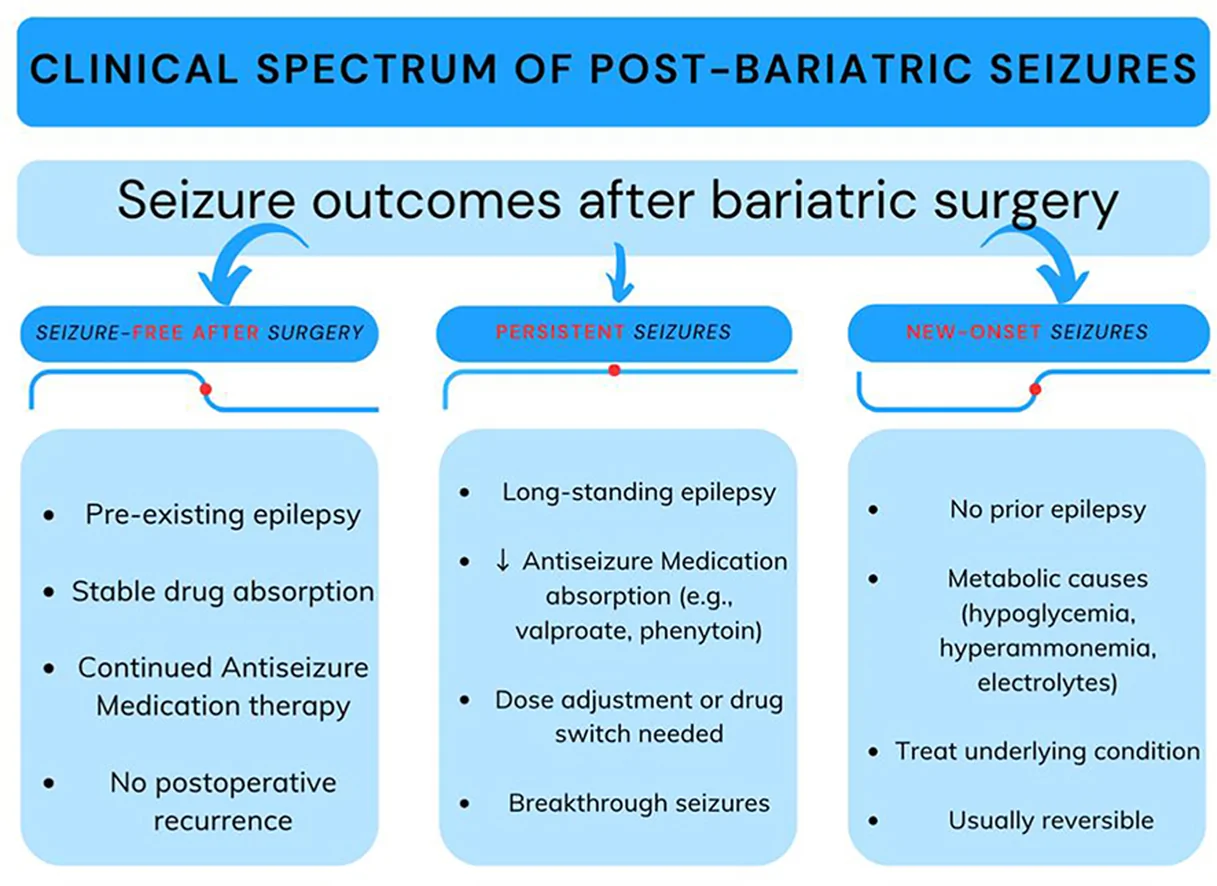

## 1 Introduction

Obesity has become increasingly prevalent worldwide and poses a major public health challenge due to its strong association with significant morbidity and mortality [1]. The lack of effective non-surgical treatments for severe obesity, combined with advances in laparoscopic techniques, has contributed to a significant increase in the number of bariatric surgeries performed worldwide [2]. Bariatric surgery encompasses a variety of procedures aimed at promoting weight loss by altering gastrointestinal anatomy and physiology, which not only facilitates substantial weight reduction but also improves comorbidities such as diabetes [3,4]. These anatomical and physiological changes can also influence drug absorption, bioavailability, and pharmacokinetics in affected patients [3]. According to the National Institute for Health and Care Excellence (NICE) guidelines, bariatric surgery may be considered for individuals with a body mass index (BMI) over 40 kg/m^2^, or between 35 and 40 kg/m^2^ if associated with significant comorbidities [4].

Neurological complications are recognized adverse events following bariatric surgery. [5]. These complications can affect the central, peripheral, and enteric nervous systems, presenting as neuropathies, cognitive changes, or other neurological disturbances. Awareness of these potential complications is crucial for clinicians to anticipate, prevent, and manage neurological issues after surgery [6,7].

While several factors are recognized to increase the risk of epilepsy, a substantial proportion of cases ranging from 44% to 69% remain of unknown etiology, suggesting the presence of additional, yet unidentified, risk factors for seizures [8]. Despite the well-documented benefits of bariatric surgery, a number of long-term neurological complications have been reported [9]. Notably, approximately 14% of bariatric surgery patients were found to be taking antiepileptic drugs prior to their procedures, highlighting the clinical importance of seizures in this population [10]. Additionally, post-prandial hyperinsulinemic hypoglycemia, a late complication particularly after Roux-en-Y gastric bypass, results from surgical anatomical changes that trigger excessive insulin release following glucose intake, which can manifest as post-meal hypoglycemia, weakness, fatigue, and in some cases, loss of consciousness or seizures [11].

Although neurological complications and metabolic disturbances after bariatric surgery are increasingly recognized, there is limited systematic investigation into seizure occurrence before and after these procedures. Understanding seizure patterns in this population is essential for optimizing perioperative care, medication management, and long-term neurological outcomes. Therefore, this study aims to investigate the current literature on the occurrence of seizures before and after bariatric surgery and to identify potential risk factors and clinical implications in this patient population.

## 2 Methods

This was a systematic review conducted according to Preferred Reporting Items for Systematic Reviews and Meta-Analyses (PRISMA) guidelines.

### 2.1 Objective

The objective of this review, framed using the PICO structure, was to evaluate patients undergoing bariatric surgery (Population) by examining how different bariatric procedures, including sleeve gastrectomy and Roux-en-Y gastric bypass (Intervention), affect seizure outcomes when compared with their preoperative neurological status (Comparison), with specific focus on postoperative seizure occurrence, timing, underlying etiologies, changes in antiseizure medication absorption or dosing, and overall neurological outcomes (Outcome).

### 2.2 Protocol and reporting

**Fig. 1:**
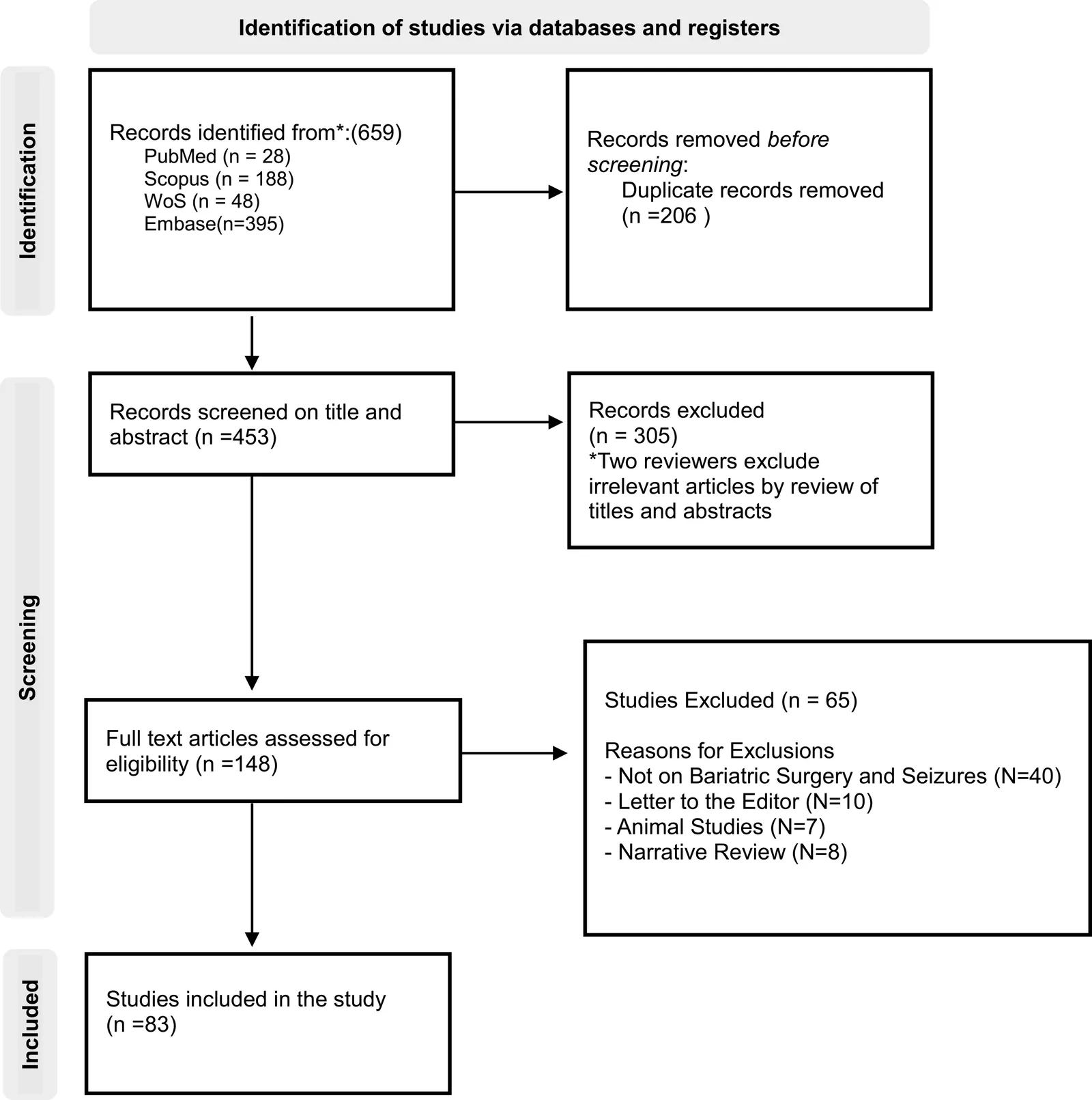
Study selection process.

This review followed the PRISMA (Preferred Reporting Items for Systematic reviews and Meta-Analyses) guidelines (Fig. 1). All data extraction sheets and quality appraisal forms were uploaded to the Open Science Framework (OSF) for transparency and accessibility (Fig. 1).

### 2.3 Data sources and search strategy

We searched PubMed, Scopus, Web of Science (WoS), and Embase from inception to the date of search. No language restrictions were applied at the query stage. The search strategy combined controlled vocabulary and free-text terms for seizures/epilepsy and bariatric surgery. All searches were completed, and data extraction was finalized on April 20, 2025 (Table 1).

**Table 1:** Search strategies.

Group	Keywords	MeSH Terms	Tiab Terms
Group 1	“Seizures” OR “seizure*" OR “Epilepsy” OR “epilep*"	“Seizures”, “Epilepsy"	“seizure*", “epilep*"
Group 2	“Bariatric Medicine” OR “Bariatrics” OR “Bariatric Surgery"	“Bariatric Medicine”, “Bariatrics”, “Bariatric Surgery"	–

### 2.4 Study selection

Two reviewers independently screened titles/abstracts and full texts. Disagreements were resolved by a third reviewer. Eligible studies included any human study (case reports, case series, cohorts, clinical trials) reporting on seizures before and/or after bariatric surgery, including seizure timing, etiology, AED changes, or clinical outcomes. Duplicates, irrelevant studies, and articles lacking clinical data were excluded.

### 2.5 Exclusion criteria

Studies were excluded if they [1] lacked sufficient clinical detail [2], were non-human studies.

### 2.6 Data extraction

Two reviewers using standardized forms independently extracted data. Extracted variables included patient demographics (age, sex, baseline BMI), comorbidities, surgery type, epilepsy history, seizure occurrence and timing, seizure etiology, AED regimens before and after surgery, and outcomes. Discrepancies were resolved by consensus or by a third reviewer.

### 2.7 Quality appraisal

The methodological quality of included studies was assessed using the Joanna Briggs Institute (JBI) critical appraisal tools, tailored to study design (case report, case series, cohort study). Quality ratings were considered in the interpretation of findings.

### 2.8 Synthesis

Due to heterogeneity in study design, populations, and outcomes, data were synthesized narratively. Where possible, descriptive statistics (e.g., distribution of seizure timing, AED adjustments, etiologies) were summarized in tables.

## 3 Results

### 3.1 Screening flow

The primary literature search produced 659 results, including 206 duplicates. After screening on title and abstract, 148 studies were found possibly relevant and underwent a full text critical appraisal, resulting in 65 exclusions. Reasons for exclusion were the following: Studies not addressing effects of metabolic bariatric surgery on Seizures (N = 40), Letter to the Editor (N = 10), Animal Studies (N = 7) and narrative Review (N = 8). The process is shown in Fig. 1 (PRISMA flow diagram) (Fig. 1).

A total of 83 studies were included in this systematic review. The majority of patients were female adults aged 30–55 years, with baseline BMI in the obese range (≥35 kg/m^2^). The two most common bariatric procedures were sleeve gastrectomy and Roux-en-Y gastric bypass (RYGB).

### 3.2 Patient characteristics

Across the included studies, patients were predominantly middle-aged adults, with a calculated mean age of 37 years. Female patients represented the majority, which reflects the demographic profile of bariatric surgery candidates (Table 5).

• Baseline BMI and changes after surgery: The mean baseline BMI was approximately 41.4 kg/m^2^, within the severely obese range. Postoperative data, although inconsistently reported, indicated significant BMI reduction after both sleeve gastrectomy and Roux-en-Y gastric bypass (RYGB) procedures.
• Common comorbidities: Where available, common comorbid conditions included type 2 diabetes mellitus, hypertension, and obstructive sleep apnea (OSA). These align with the typical medical background of bariatric surgery candidates and may influence seizure outcomes and pharmacological responses.

### 3.3 Preoperative seizures without recurrence after surgery

In several studies, patients with established epilepsy (treated with lamotrigine, valproate, or levetiracetam) experienced no seizure recurrence postoperatively [12]. Stable pharmacokinetics and continued drug therapy contributed to favorable outcomes (Table 2).

**Table 2:** Summary of reported seizure history and management **before** bariatric surgery.

Author/Year	Location	Bariatric Surgery type	Outcomes
Schoretsanitis-2024 [12]	Norway	Roux-en-Y gastric bypass, sleeve gastrectomy	Epileptic syndromes on lamotrigine or valproate continued treatment post-surgery with dose adjustments.
Sargsyan-2023 [13]	UK	Sleeve gastrectomy, Gastric bypass, Gastric band	Patients on unspecified antiepileptic drugs remained mostly seizure-free post-surgery with no new epilepsy type, continued baseline therapy, and no reported side effects.
Rives-Lange-2023 [14]	France	Gastric bypass and sleeve gastrectomy	Not epilepsy; but children of post-MBS pregnancies had lower rates of febrile convulsions
Nagarur-2023 [15]	USA	Roux-en-Y gastric bypass	Long-standing seizure disorder continued therapy; later myoclonus was due to hyperammonemia, not epilepsy.
Margolin-2023 [16]	Israel	various types of bariatric surgeries	Epilepsy on carbamazepine or levetiracetam recurred in some patients within a year post-surgery, with no change in therapy.
Holländer-2023 [17]	Germany	Roux-en-Y gastric bypass, conversion to sleeve gastrectomy with Roux limb as Henley-Longmire interposition	Seizures linked to severe PBH resolved after surgery with improved glucose levels.
Dunn-2023 [17]	USA	Roux-en-Y gastric bypass	PBH-related seizures resolved after TORe.
McDermond-2022 [18]	USA	Gastric bypass	Seizure disorder controlled on duloxetine, quetiapine, and lamotrigine, with stable therapy post-surgery.
Wallerstedt-2021 [19]	Sweden	Gastric bypass, Sleeve gastrectomy	Gabapentin, lamotrigine, and pregabalin were continued post-surgery with monitored serum levels, but no seizure data.
Porat-2021 [20]	Israel	Roux-en-Y gastric bypass, sleeve gastrectomy	Long-term epilepsy on lamotrigine continued post-surgery, with potential absorption issues.
Jain-2021 [21]	USA	Roux-en-Y gastric bypass	Seizure disorder showed no recurrence after gastric bypass, though hypoglycemia worsened with glucose.
Bardaro-2017 [22]	USA	Laparoscopic sleeve gastrectomy	No seizures reported post-surgery
San Martin-2016 [23]	Brazil	Laparoscopic sleeve gastrectomy	PCT-related seizures resolved after acetazolamide, weight loss, and lifestyle changes.
Koutsavlis-2015 [24]	UK	Sleeve gastrectomy	Temporal lobe epilepsy remained controlled post-op, but carbamazepine toxicity required dose reduction.
Heinberg-2014 [25]	USA	Roux-en-Y gastric bypass	Seizure disorder since infancy remained well controlled on Depakote before and after surgery.
Spritzer-2013 [26]	USA	Roux-en-Y gastric bypass	Two patients with pre-existing seizure disorders had no worsening or new epilepsy after RYGB.
Adamo-2013 [27]	UK	Laparoscopic sleeve gastrectomy	All 11 epilepsy patients on medication had stable control post-surgery, except one perioperative seizure from missed dose.
Rivas-2009 [28]	Mexico	Laparoscopic Roux-en-Y Gastric Bypass	Partial seizures were reported pre-surgery, but post-surgery seizure outcomes were not described.

**Abbreviations:** RYGB = Roux-en-Y Gastric Bypass, LRYGB = Laparoscopic Roux-en-Y Gastric Bypass, SG = Sleeve Gastrectomy, SADJ-S = Single Anastomosis Duodeno-Jejunal Bypass with Sleeve, TORe = Transoral Outlet Reduction, eTOR = Endoscopic Transoral Outlet Reduction, AEDs = Antiepileptic Drugs, PRES = Posterior Reversible Encephalopathy Syndrome, LOC = Loss of Consciousness, BG = Blood Glucose, PCT = Porphyria Cutanea Tarda, PBH = Post-Bariatric Hypoglycemia, **RFS =** Refeeding Syndrome, **HCC =** Hepatocellular Carcinoma, HAART = Highly Active Antiretroviral Therapy, ICU = Intensive Care Unit, HR = Hazard Ratio, CI = Confidence Interval, DM = Diabetes Mellitus, OSA = Obstructive Sleep Apnoea.

### 3.4 Persistent seizures before and after surgery

Some patients with long-standing epilepsy or structural causes (e.g., post-stroke epilepsy) continued to experience seizures despite surgery [[29], [30], [31]]. In this group, inadequate absorption of valproate and phenytoin after RYGB was a common reason for recurrence, requiring dose escalation or switch to lamotrigine (Table 3). Several reports describe patients who had epilepsy prior to bariatric surgery and continued to experience seizures postoperatively. These individuals were typically middle-aged adults (40–60 years) with both male and female representation, undergoing predominantly Roux-en-Y gastric bypass (RYGB)(Table 3).

**Table 3:** Summary of reported seizure history and management **before and after** bariatric surgery.

Author	Location	Bariatric Surgery type	Outcomes
Wong;-2015 [32]	UK	N/A	Hypoglycemia-related seizures decreased significantly after surgical reversal.
Wolter-2012 [29]	Germany	Laparoscopic Roux-en-Y gastric bypass	Epilepsy since adolescence on valproic acid showed reduced drug absorption post-op, raising seizure risk.
Clemmons-2012 [33]	USA	Roux-en-Y gastric bypass	Twelve patients with pre-existing epilepsy remained stable post-op, while five developed new but non-intractable epilepsy.
Woods-2011 [30]	Ireland	Roux-en-Y Gastric Bypass → Reversal with Sleeve Gastrectomy	Post-stroke epilepsy, seizure-free for 5 years, recurred after RYGB but was controlled after reversal.
Pournaras-2011 [31]	UK	Laparoscopic Roux-en-Y Gastric Bypass	Generalized epilepsy well-controlled for 30 years relapsed after RYGB, requiring therapy changes before regaining seizure control.

Rodrigues-2010 [34]	USA	Gastric bypass	Recurrent generalized seizures developed years after gastric bypass, managed alongside dysautonomia and hemochromatosis.
Triplett-2021 [35]	USA	Roux-en-Y gastric bypass, sleeve gastrectomy	In 44 epilepsy patients on various AEDs, 5 had new seizures within 6 months and 9 had long-term worsening post-surgery, requiring dose adjustments.
Porat D-2022 [20]	Israel	Sleeve gastrectomy (SG)	4/8 had decreased CBZ levels; 2 had worsening disease (1 epilepsy, 1 trigeminal neuralgia); Patient 1 → uncontrolled epilepsy → lobectomy 13 months post-SG; others required increased CBZ dosage; recommendation: TDM & careful monitoring

**Abbreviations:** RYGB = Roux-en-Y Gastric Bypass, LRYGB = Laparoscopic Roux-en-Y Gastric Bypass, SG = Sleeve Gastrectomy, SADJ-S = Single Anastomosis Duodeno-Jejunal Bypass with Sleeve, TORe = Transoral Outlet Reduction, eTOR = Endoscopic Transoral Outlet Reduction, AEDs = Antiepileptic Drugs, PRES = Posterior Reversible Encephalopathy Syndrome, LOC = Loss of Consciousness, BG = Blood Glucose, PCT = Porphyria Cutanea Tarda, PBH = Post-Bariatric Hypoglycemia, **RFS =** Refeeding Syndrome, **HCC =** Hepatocellular Carcinoma, HAART = Highly Active Antiretroviral Therapy, ICU = Intensive Care Unit, HR = Hazard Ratio, CI = Confidence Interval, DM = Diabetes Mellitus, OSA = Obstructive Sleep Apnoea.

Overall, patients who continued to seize after surgery were those with:

1. Pre-existing epilepsy of long duration (often >20 years).
2. Pharmacokinetic challenges with antiepileptic drugs post-RYGB.
3. Persistent or secondary causes such as hypoglycemia or stroke sequelae.

This highlights the need for close therapeutic drug monitoring, consideration of surgery type, and individualized AED adjustments in patients with epilepsy undergoing bariatric procedures.

### 3.5 New-onset seizures after surgery

Multiple reports described new seizures in patients without prior epilepsy, with onset ranging from hours post-op to 10 years later. Etiologies were mostly secondary and metabolic, including hypoglycemia, hyperammonemia, torsades de pointes, and electrolyte disturbances [[36], [37], [38], [39]]. Management targeted the underlying cause, with generally favorable outcomes (Table 4). Overall, bariatric surgery exerts heterogeneous effects on seizure outcomes, ranging from long-term seizure freedom to recurrence or new-onset episodes linked to systemic complications. These findings underscore the importance of individualized patient care, including close monitoring of antiseizure drug levels, nutritional status, and metabolic health, both in the immediate postoperative period and long-term follow-up. Clinicians should remain vigilant to distinguish between primary epilepsy and secondary seizure causes in this unique patient population (Table 5, Table 6).

**Table 4:** Summary of reported seizure and management **after** bariatric surgery.

Author	Location	Bariatric Surgery type	Outcomes
Kiani-2025 [36]	USA	Sleeve gastrectomy	A seizure-like episode 3 months post-sleeve gastrectomy was due to Torsades de Pointes and syncope, not epilepsy.
Zeghlache-2024 [37]	USA	Roux-en-Y gastric bypass	Seizures 7 years post-RYGB were due to nonhepatic hyperammonemic encephalopathy, not primary epilepsy.
Thomas-2024 [38]	USA	Gastric sleeve	Postprandial seizures after surgery improved with diazoxide and dietary modification.
Shaikh-2024 [40]	Qatar	sleeve gastrectomy	developed acute symptomatic PRES-related seizures within hours post-op, treated with levetiracetam.
Prifti-2024 [39]	USA	Roux-en-Y Gastric Bypass	Ten years post-RYGB, Case 1 had acute hyperammonemia-related seizures treated with AEDs, while Case 2 likely had metabolic encephalopathy without chronic epilepsy.
Park-2024 [41]	Australia	Roux-en-Y Gastric Bypass	Seizures 3 years post-RYGB were secondary to neuroglycopenia and managed metabolically, not with AEDs.
Ntelis-2024 [42]	USA	Roux-en-Y Gastric Bypass	Late post-surgery seizures were due to hypoglycemia, managed with acarbose, diet, and octreotide-to-lanreotide transition.
Mallik-2024 [43]	UK	Roux-en-Y Gastric Bypass	Seizures were linked to post-bariatric hypoglycemia and managed with metabolic treatments, not AEDs.
Lloyd-2024 [44]	USA	Roux-en-Y gastric bypass	Suspected acute myoclonic status epilepticus occurred years after RYGB, treated with levetiracetam.
Jou-2024 [45]	USA	Roux-en-Y gastric bypass	Nonconvulsive status epilepticus developed years after RYGB following duodenostomy/pyloroplasty, treated with levetiracetam.
Habbsa-2023 [46]	USA	Roux-en-Y gastric bypass	PBH-related seizures began 12 months post-surgery, worsened over time, and were managed with a gastrostomy tube.
Torcida Sedano-2022 [47]	Belgium	Gastric bypass	Generalized tonic–clonic seizures progressed to refractory status epilepticus 6 months post-bypass, treated with multiple AEDs.
Paknikar-2022 [48]	USA	Roux-en-Y gastric bypass	10 years post-RYGB
Margolin-2022 [49]	Israel	Sleeve gastrectomy, Roux-en-Y gastric bypass	Among three patients, seizures worsened after SG in two and remained controlled after RYGB in one, with levetiracetam dose increases as needed.
Edelson-2022 [50]	USA	Roux-en-Y gastric bypass	Hypoglycemia-related seizures years after RYGB resolved after eTOR, allowing discontinuation of acarbose, octreotide, and glucagon.
Antaya-2022 [51]	Canada	Roux-en-Y gastric bypass (RYGB), sleeve gastrectomy (SG)	Bariatric surgery was associated with new-onset epilepsy and increased risk compared to non-surgical patients.
Sundström-2021 [52]	Sweden	Gastric bypass	Seizures occurred 8 years post-surgery due to refeeding syndrome.
Sjöholm-2021 [53]	Sweden	Roux-en-Y gastric bypass, vertical banded gastroplasty, banding	Within 6 months post-surgery, 5 non-diabetic patients developed hypoglycemia-related seizures.
Patel-2021 [54]	USA	Roux-en-Y gastric bypass	Myoclonic status epilepticus due to hyperammonemia occurred 10 days post-RYGB, treated with AEDs.
Omar-2021 [55]	USA	Roux-en-Y gastric bypass	Seizures from hyperammonemia occurred 3 years post-surgery, requiring ICU care and treatment with lactulose, rifaximin, zinc, and arginine.
McManus-2021 [56]	USA	Roux-en-Y gastric bypass	Hypoglycemia-related seizures occurred 10 years post-bypass, worsened by glucose, and managed with diet and acarbose.
Cardona-Gonzalez-2021 [56]	Puerto Rico	Roux-en-Y gastric bypass	Seizures due to PRES occurred within days after surgery, managed with prophylaxis.
Vadhar-2020 [57]	USA	Roux-en-Y gastric bypass	Hyperammonemia-related seizures occurred 3 months post-surgery.
Farahmand-2020 [58]	USA	Gastric bypass	PBH-related seizures began 1 year post-surgery, treated with Levetiracetam and later diazoxide.
El Sheikh-2020 [59]	Middle Eastern	Sleeve gastrectomy	Hyperammonemia-related seizures occurred 2 months post-surgery, requiring sedation and AED treatment.
Bhutta-2020 [60]	USA	Roux-en-Y gastric bypass	Seizures occurred post-surgery as a complication of critical illness (sepsis, respiratory failure, hypotension), not epilepsy.
Bergagnini-2020 [61]	USA	Roux-en-Y gastric bypass	Seizures from hyperammonemia occurred 4 months after RYGB revision, progressing to status epilepticus and treated with sedation and AEDs.
Sen-2019 [62]	USA	Gastric sleeve	Post-bariatric surgery seizures from acquired urea cycle disorder with hyperammonemia occurred during metastatic HCC treatment, managed with levetiracetam.
Leap-2019 [63]	USA	Gastric bypass	Acute seizures post-bypass were due to magnesium toxicity from Epsom salt ingestion, treated with levetiracetam and hemodialysis.
Jones-2019 [64]	USA	Laparoscopic sleeve gastrectomy	A known preoperative seizure disorder had a postoperative seizure that resolved.
Franca Tornincasa-2019 [65]	Brazil	Roux-en-Y gastric bypass	Post-RYGB seizures were linked to hypocalcemia and managed with corrective treatment and surgery.
Derek Yeung-2019 [66]	UK	One anastomosis gastric bypass	Post-surgery seizures were due to hypocalcemia, not epilepsy.
Conaty;-2019 [67]	USA	Roux-en-Y gastric bypass	Post-bypass seizures were due to hypoglycemia and treated with levetiracetam.
Grogg-2018 [68]	USA	Roux-en-Y gastric bypass and small bowel resection	Seizures from hyperammonemia occurred 15 years post-RYGB (5 years post-bowel resection) and resolved with treatment including levetiracetam.
De Oliveira-2018 [69]	Brazil	Roux-en-Y gastric bypass	Hypoglycemia-related seizures occurred 1.5 years post-surgery and were managed with acarbose and diazoxide.
Brown-2018 [70]	USA	Roux-en-Y gastric bypass	Reactive hypoglycemia caused seizures 1.5 years post-bypass, managed by treating hypoglycemia.
Tsao-2017 [71]	Taiwan	Billroth II gastrectomy (Patient 1), Billroth II (Patient 2)	Post-surgery seizures due to Wernicke's encephalopathy resolved with thiamine supplementation.
Silva-2016 [72]	Portugal	Roux-en-Y gastric bypass	Seizures from postprandial hypoglycemia occurred 16 months post-surgery, partially managed with acarbose and diet, with surgery considered.
Curell-2016 [73]	Spain	SADI-S, converted to SADJ-S or Roux-en-Y gastric bypass	One patient developed status epilepticus from metabolic disorders post-surgery, requiring ICU care and nutritional management.
Parampalli-2015 [74]	UK	Roux-en-Y gastric bypass	Seizures from malnutrition and micronutrient imbalance resolved after gastric bypass reversal.
Kromas-2015 [75]	USA	Roux-en-Y gastric bypass	Hyperammonemia-related seizures developed 2 years post-surgery and were managed with lactulose, sodium benzoate, and protein restriction.
Hahn-2015 [76]	USA	Roux-en-Y gastric bypass	Hyperammonemia-related seizures with status epilepticus occurred 11 years post-RYGB, treated with phenobarbital.
Atla-2015 [77]	USA	Roux-en-Y gastric bypass	Hypoglycemia-related seizures occurred 5 years post-RYGB after 2 years of worsening episodes, managed with diazoxide and diet.
Wong-2014 [78]	UK	Roux-en-Y gastric bypass (RYGB)	Hypoglycemia-related seizures after RYGB improved after reversal, decreasing from daily to weekly episodes.
Safdieh-2014 [79]	USA	Gastric bypass	New seizures 5 years post-bypass were due to hypoglycemia and later hepatic encephalopathy, managed with lorazepam and metabolic treatments.
Rao-2014 [80]	USA	Roux-en-Y gastric bypass	Recurrent hypoglycemia-related seizures persisted within 9 months post-RYGB despite medical therapy, requiring G-tube feeds and eventual reversal.
Narla-2014 [81]	USA	Roux-en-Y gastric bypass	Severe hypoglycemia (BG 30–40 mg/dL) caused daily seizures 4 years post-bypass, managed with diazoxide, octreotide, and later tumor surgery.
Geron-2014 [82]	Israel	Laparoscopic sleeve gastrectomy	A single seizure 9 months post-surgery due to B12 deficiency resolved with supplementation.
Lee-2013 [83]	USA	Roux-en-Y gastric bypass	Post-RYGB hypoglycemia caused persistent seizures despite reversal and medical therapy, with weekly episodes or LOC in two patients.
Woods-2012 [84]	Ireland	Roux-en-Y gastric bypass	One patient developed seizures from severe recurrent hypoglycemia post-RYGB, managed with hypoglycemia treatment and reversal.
Rabiee-2010 [85]	USA	Roux-en-Y Gastric Bypass	New-onset seizures 3 years post-RYGB were due to profound hypoglycemia, managed with octreotide and later distal pancreatectomy.
Marsk-2010 [86]	Sweden	Roux-en-Y Gastric Bypass and vertical banded gastroplasty and gastric banding	Post-RYGB, risk increased (HR 3.0 for epilepsy; HR 7.3 for seizures), with median onset 2.7 years post-op.
Naslund-2009 [87]	Sweden	Roux-en-Y Gastric Bypass	After RYGB, risks increased (HR 3.9 epilepsy, 8.3 seizures, 4.9 syncope, 2.3 confusion), with median follow-up 2.3 years.
Fazylov-2007 [88]	USA	Roux-en-Y Gastric Bypass	New-onset seizures occurred 6 months after LRYGB, requiring anticonvulsants alongside HAART.
Choi-2004 [89]	USA	Roux-en-Y Gastric Bypass	A single generalized seizure with aura occurred 4 months post-RYGB after stroke, treated with fosphenytoin then levetiracetam.
Salas-Salvadó-2000 [90]	Spain	Gastroplasty + Y-Roux gastrojejunum bypass	Case 2 developed generalized seizures 1 year post-bypass from thiamine deficiency/Wernicke's, treated with hydantoins, phenobarbital, and thiamine.

**Abbreviations:** RYGB = Roux-en-Y Gastric Bypass, LRYGB = Laparoscopic Roux-en-Y Gastric Bypass, SG = Sleeve Gastrectomy, SADJ-S = Single Anastomosis Duodeno-Jejunal Bypass with Sleeve, TORe = Transoral Outlet Reduction, eTOR = Endoscopic Transoral Outlet Reduction, AEDs = Antiepileptic Drugs, PRES = Posterior Reversible Encephalopathy Syndrome, LOC = Loss of Consciousness, BG = Blood Glucose, PCT = Porphyria Cutanea Tarda, PBH = Post-Bariatric Hypoglycemia, **RFS =** Refeeding Syndrome, **HCC =** Hepatocellular Carcinoma, HAART = Highly Active Antiretroviral Therapy, ICU = Intensive Care Unit, HR = Hazard Ratio, CI = Confidence Interval, DM = Diabetes Mellitus, OSA = Obstructive Sleep Apnoea.

**Table 5:** Patterns of seizure presentation and management in bariatric surgery patients.

Group	Age/Sex (Typical)	Surgery Type	Comorbidities	Timing of Seizures	Type & Etiology	AED/Management	Outcome
Pre-op seizures, no recurrence	30s–40s, mostly female	Sleeve, RYGB	Obesity-related (DM, OSA)	No seizures during follow-up (≤5 yrs)	Primary epilepsy controlled	Continued lamotrigine/valproate/levetiracetam	Stable seizure-free
Seizures before & after	40s–60s, both sexes	Mostly RYGB	Long-standing epilepsy, stroke	Months–years post-op	Breakthrough epilepsy due to poor AED absorption	Dose ↑ or switch (phenytoin → lamotrigine, ↑phenobarbital)	Variable; some improved after reversal
New-onset seizures	30s–50s, mostly female	Sleeve & RYGB (late cases RYGB)	Metabolic complications	Hours → 10 years post-op	Secondary: hypoglycemia, hyperammonemia, arrhythmia	Correct underlying cause ± short-term AED	

**Abbreviations:** RYGB = Roux-en-Y Gastric Bypass, LRYGB = Laparoscopic Roux-en-Y Gastric Bypass, SG = Sleeve Gastrectomy, SADJ-S = Single Anastomosis Duodeno-Jejunal Bypass with Sleeve, TORe = Transoral Outlet Reduction, eTOR = Endoscopic Transoral Outlet Reduction, AEDs = Antiepileptic Drugs, PRES = Posterior Reversible Encephalopathy Syndrome, LOC = Loss of Consciousness, BG = Blood Glucose, PCT = Porphyria Cutanea Tarda, PBH = Post-Bariatric Hypoglycemia, **RFS =** Refeeding Syndrome, **HCC =** Hepatocellular Carcinoma, HAART = Highly Active Antiretroviral Therapy, ICU = Intensive Care Unit, HR = Hazard Ratio, CI = Confidence Interval, DM = Diabetes Mellitus, OSA = Obstructive Sleep Apnoea.

**Table 6:** Timing patterns and etiologies of seizure onset after bariatric surgery.

Time of Onset	Approximate Frequency	Typical Clinical Context	Underlying Etiology
Several years post-op	Most frequent	Chronic nutritional deficiency, delayed complications	Malabsorption, thiamine deficiency, hyperammonemia, long-term AED Pharmacokinetic changes
Several months post-op	Second most common	Rapid weight loss, metabolic changes, AED adjustment	Altered absorption of AEDs (↓Valproate, ↑Lamotrigine), hypoglycemia, nutritional deficits
Weeks to days post-op	Less common but important	Immediate post-op phase	Acute metabolic/electrolyte imbalance, thiamine deficiency/Wernicke's encephalopathy
Unspecified	Frequent	Case reports with insufficient details	Not clearly reported

**Abbreviations:** RYGB = Roux-en-Y Gastric Bypass, LRYGB = Laparoscopic Roux-en-Y Gastric Bypass, SG = Sleeve Gastrectomy, SADJ-S = Single Anastomosis Duodeno-Jejunal Bypass with Sleeve, TORe = Transoral Outlet Reduction, eTOR = Endoscopic Transoral Outlet Reduction, AEDs = Antiepileptic Drugs, PRES = Posterior Reversible Encephalopathy Syndrome, LOC = Loss of Consciousness, BG = Blood Glucose, PCT = Porphyria Cutanea Tarda, PBH = Post-Bariatric Hypoglycemia, **RFS =** Refeeding Syndrome, **HCC =** Hepatocellular Carcinoma, HAART = Highly Active Antiretroviral Therapy, ICU = Intensive Care Unit, HR = Hazard Ratio, CI = Confidence Interval.

### 3.6 Comparison of the three groups

When comparing the three subgroups, several patterns emerge (Table 7, Table 8):

1. **Age and Sex**: Across all groups, most patients were female adults in their 30s–50s, reflecting the general demographic of bariatric surgery. However, persistent epilepsy cases (before-and-after group) included some older adults (up to 60s) with long-standing epilepsy.
2. **Type of Surgery**: Both sleeve gastrectomy and Roux-en-Y gastric bypass (RYGB) were represented in all groups. Patients who became seizure-free were often managed successfully after either RYGB or sleeve. In contrast, RYGB was overrepresented in patients with persistent or new seizures, particularly due to its stronger effect on drug absorption and metabolic complications.
3. **Comorbidities**: Those who became seizure-free often had obesity-related metabolic comorbidities that improved after weight loss (e.g., insulin resistance, sleep apnea). Persistent seizure patients frequently had chronic neurological conditions such as long-standing epilepsy or post-stroke epilepsy. New-onset seizure cases were linked to surgery-related metabolic complications including hypoglycemia, hyperammonemia, and arrhythmias.
4. **Timing of Seizures**:
  o Seizure-free group: no recurrence during follow-up (up to 5 years).
  o Persistent group: recurrence ranged from months to years, often when drug absorption became inadequate or underlying pathology (stroke) persisted.
  o New-onset group: onset ranged from hours post-op to 10 years after surgery, depending on the underlying systemic trigger.
5. **Type and Etiology of Seizures**:
  o Seizure-free: preoperative epilepsy (generalized or focal) remained stable after surgery.
  o Persistent: recurrent epilepsy due to drug malabsorption (valproate, phenytoin), long duration of disease, or vascular etiology.
  o New-onset: secondary seizures due to metabolic derangements (hypoglycemia, hyperammonemia, torsades de pointes).
6. **Drugs and Management**:
  o Seizure-free: continued lamotrigine, valproate, or levetiracetam, with stable levels.
  o Persistent: required dose escalation or switch (e.g., phenytoin → lamotrigine, higher phenobarbital).
  o New-onset: mainly managed by treating underlying cause (diazoxide for hypoglycemia, lactulose/rifaximin for hyperammonemia, pacemaker for arrhythmia), with short-term AED use when needed.
7. **Outcomes**:
  o Seizure-free: durable remission.
  o Persistent: partial or full control achievable with drug adjustment or surgery reversal in some cases.
  o New-onset: outcomes generally favorable once the systemic trigger was corrected; long-term epilepsy rarely developed.

**Table 7:** Comparative table of three patient groups.

Category	Seizure-Free After Surgery	Seizures Before & After Surgery	New-Onset Seizures After Surgery
Age & Sex	Mostly 30s–50s, female predominance	40s–60s, both sexes, often long history	30s–50s, mostly female
Surgery Type	Sleeve & RYGB (both effective)	Predominantly RYGB	Both sleeve & RYGB; late cases often RYGB
Comorbidities	Obesity-related (diabetes, sleep apnea) improved post-op	Chronic epilepsy, stroke history	Metabolic/secondary causes: hypoglycemia, hyperammonemia, arrhythmia
Timing	No recurrence during follow-up (up to 5 yrs)	Months–years after surgery (esp. drug malabsorption)	Hours to 10 years post-op
Seizure Type & Etiology	Pre-op epilepsy controlled post-op	Long-standing epilepsy, drug absorption issues, vascular epilepsy	Secondary/metabolic seizures (hypoglycemia, ammonia, torsades)
Drugs & Management	Continued lamotrigine, valproate, levetiracetam	Dose escalation or switch (phenytoin → lamotrigine, ↑phenobarbital)	Treat underlying cause; short-term AED (levetiracetam, unspecified)
Outcome	Remission	Variable; some controlled after drug change or reversal	Favorable if cause corrected; rarely chronic epilepsy

**Abbreviations:** RYGB = Roux-en-Y Gastric Bypass, LRYGB = Laparoscopic Roux-en-Y Gastric Bypass, SG = Sleeve Gastrectomy, SADJ-S = Single Anastomosis Duodeno-Jejunal Bypass with Sleeve, TORe = Transoral Outlet Reduction, eTOR = Endoscopic Transoral Outlet Reduction, AEDs = Antiepileptic Drugs, PRES = Posterior Reversible Encephalopathy Syndrome, LOC = Loss of Consciousness, BG = Blood Glucose, PCT = Porphyria Cutanea Tarda, PBH = Post-Bariatric Hypoglycemia, **RFS =** Refeeding Syndrome, **HCC =** Hepatocellular Carcinoma, HAART = Highly Active Antiretroviral Therapy, ICU = Intensive Care Unit, HR = Hazard Ratio, CI = Confidence Interval.

**Table 8:** Abbreviations.

Abbreviation	Full Term	Abbreviation	Full Term
RYGB	Roux-en-Y Gastric Bypass	**PCT**	Porphyria Cutanea Tarda
LRYGB	Laparoscopic Roux-en-Y Gastric Bypass	**PBH**	Post-Bariatric Hypoglycemia
SG	Sleeve Gastrectomy	**RFS**	Refeeding Syndrome
SADJ-S	Single Anastomosis Duodeno-Jejunal Bypass with Sleeve	**HCC**	Hepatocellular Carcinoma
TORe	Transoral Outlet Reduction		
eTOR	Endoscopic Transoral Outlet Reduction	**HAART**	Highly Active Antiretroviral Therapy
AEDs	Antiepileptic Drugs	**ICU**	Intensive Care Unit
PRES	Posterior Reversible Encephalopathy Syndrome	**HR**	Hazard Ratio
LOC	Loss of Consciousness	**CI**	Confidence Interval
BG	Blood Glucose		

## 4 Discussion

### 4.1 Overall summary of findings

Across the included reports, most patients were middle-aged adults, predominantly women, with baseline BMI in the obese range (≥35–40 kg/m^2^) [12,[36], [37], [38],^40^]. Common comorbidities such as metabolic disorders were present, although reporting was inconsistent.

Epilepsy before surgery was less frequently documented, with antiepileptic drugs most often including lamotrigine or valproate [12]. In most patients with pre-existing epilepsy, seizure control was stable before the procedure.

Following bariatric surgery, outcomes were heterogeneous. New-onset seizures were observed in some patients within weeks to months [36,40], and in rare cases years after surgery [37]. For patients with prior epilepsy, seizure frequency often remained stable, though breakthrough or acute symptomatic seizures occurred in some cases [40].

Medication adjustments were reported, especially after Roux-en-Y gastric bypass (RYGB). Lamotrigine exposure modestly increased, while valproate levels decreased, sometimes requiring dose modifications [12]. In some cases, antiepileptic drugs were discontinued or switched [36,37].

Differences by surgery type were noted: sleeve gastrectomy was more commonly associated with early metabolic-related seizures [38,40], while RYGB was linked to pharmacokinetic changes in antiepileptic drugs [12].

Overall, most patients maintained or improved seizure control postoperatively, although a minority experienced worsening, underscoring the need for individualized monitoring and therapeutic drug (Table 5).

### 4.2 Effect of bariatric surgery on seizure outcomes

Bariatric surgery has become an increasingly common intervention for obesity, but its impact on seizure outcomes remains a subject of debate, with evidence pointing to both beneficial and adverse effects. The literature reveals three main patterns: patients with pre-existing epilepsy who remain seizure-free after surgery, those with persistent or recurrent seizures, and those who develop new-onset seizures postoperatively.

For patients with epilepsy prior to surgery, outcomes are often favorable. Sargsyan et al. (2023) reported that many patients remained seizure-free for up to five years postoperatively, suggesting a stabilizing effect of bariatric surgery on seizure control [13]. Similarly, Schoretsanitis et al. (2024) noted pharmacokinetic changes in antiepileptic drugs such as lamotrigine and valproate but without significant seizure exacerbation [12]. However, other studies, such as Margolin et al. (2023), highlighted decreased carbamazepine serum levels in some cases, resulting in breakthrough seizures, while delayed metabolic or nutritional complications have also been linked to seizure recurrence [15,16].

In patients with persistent or recurrent seizures, the evidence is mixed. Clemmons and Cascino (2012) found no exacerbation of seizures among 1542 RYGB patients with epilepsy, though a small subset developed new-onset epilepsy [33]. In contrast, case reports by Pournaras et al. (2011) and Wolter et al. (2012) demonstrated impaired absorption of phenytoin and valproic acid, leading to recurrent seizures unless dose adjustments were made [29,31]. Similarly, Woods et al. (2011) described seizure recurrence two years after RYGB that resolved after reversal, while Wong and Dexter (2015) reported hypoglycemia-induced seizures that also improved with reversal [30,32]. These findings suggest that while bariatric surgery does not universally worsen seizure control, altered drug metabolism, nutritional deficiencies, and metabolic complications can increase risk.

Finally, in patients without prior epilepsy, seizures after bariatric surgery often arise from secondary causes rather than primary epilepsy. Kiani et al. (2025) described seizures three months after sleeve gastrectomy, later attributed to Torsades de Pointes and corrected with pacemaker implantation [36]. Zeghlache et al. (2024) reported late-onset seizures seven years post-RYGB caused by non hepatic hyperammonemia, while Thomas et al. (2024) described postprandial hypoglycemia-induced seizures successfully managed with diazoxide and diet [37,38]. Shaikh et al. (2024) found acute symptomatic seizures linked to PRES in a small case series, and Prifti et al. (2024) reported hyperammonemia and hepatic dysfunction as seizure triggers a decade after RYGB(19, 20).

### 4.3 Patients with seizures before surgery but not after

Across multiple reports, a notable subgroup of patients with preoperative epilepsy achieved seizure freedom after bariatric surgery. In the prospective pharmacokinetic cohort by Schoretsanitis et al. (2024), eight patients (six on lamotrigine, two on valproate) with established epilepsy remained seizure-free throughout follow-up, despite modest pharmacokinetic changes in drug levels. Similarly, in the retrospective review by Sargsyan et al. (2023), 7 of 11 patients with a history of epilepsy did not experience postoperative seizures during up to 5 years of observation, with continued baseline therapy and no significant adverse events [12,13](Table 2).

Findings were echoed in larger database studies. Rives-Lange et al. (2023), in a nationwide French cohort of >53,000 women, included patients with pre-surgical seizure disorders; long-term follow-up suggested no excess seizure recurrence after either gastric bypass or sleeve gastrectomy, supporting the safety of surgery in this population [14]. Margolin et al. (2023) also reported cases where preoperative epilepsy stabilized postoperatively under continued carbamazepine or levetiracetam therapy [16].

Reasons for postoperative seizure freedom appear multifactorial. In several cases, seizures prior to surgery were attributed not solely to refractory epilepsy but also to obesity-associated comorbidities (e.g., metabolic syndrome, sleep apnea, glucose dysregulation) that may have lowered seizure threshold. Sustained weight loss after surgery whether via Roux-en-Y gastric bypass or sleeve gastrectomy likely alleviated these systemic risk factors. Moreover, most patients remained on antiepileptic drugs with favorable absorption profiles post-surgery (lamotrigine, levetiracetam, valproate), ensuring stable control.

Demographically, the majority were female adults in their 30s–50s, aligning with the typical bariatric surgery population. Taken together, these findings indicate that for some patients, bariatric surgery not only improves metabolic health but may also contribute to seizure remission, provided that antiepileptic drugs are maintained and monitored.

### 4.4 Patients with persistent or recurrent seizures before and after surgery

The underlying causes of seizures varied. In some patients, seizures before surgery were secondary to metabolic disorders such as hypoglycemia; however, despite weight loss, episodes persisted when hypoglycemia remained unresolved [32]. Others had long-standing primary epilepsy, often generalized or post-stroke, which was not fully controlled preoperatively and therefore recurred afterward [[29], [30], [31]].

Medication-related issues played a major role. Reduced absorption of antiepileptic drugs particularly valproic acid and phenytoin was observed after RYGB, leading to subtherapeutic serum concentrations and breakthrough seizures [29,31]. Even patients who had remained seizure-free for decades relapsed after surgery due to inadequate drug bioavailability [31]. In some cases, doubling the drug dose or switching to alternative agents (e.g., lamotrigine) was necessary to regain control.

Importantly, structural or vascular etiologies also contributed: one case involved post-stroke epilepsy with seizure recurrence two years post-RYGB, only controlled after surgical reversal [30]. This suggests that for some patients, bariatric surgery itself is not the trigger, but rather it interacts with pre-existing neurological vulnerabilities.

### 4.5 Patients with new-onset seizures after surgery

New-onset seizures were documented in several case reports and small series, typically affecting female patients in their 30s–50s who underwent either sleeve gastrectomy or Roux-en-Y gastric bypass (RYGB). The timing of onset was heterogeneous: some developed seizures within hours to days postoperatively, while others presented after months or even years to a decade following RYGB [36,37,39,40] (Table 4) (Table 6)

The underlying causes were diverse and often secondary to metabolic or systemic complications rather than primary epilepsy. Reported etiologies included cardiac arrhythmia with torsades de pointes, non-hepatic hyperammonemia, postprandial hypoglycemia, and acute symptomatic seizures associated with metabolic stress [[36], [37], [38],^40^]. In a few cases, late-onset hyperammonemia and hypoglycemia more than 10 years post-RYGB were described [39].

Management depended on the underlying trigger. Rather than long-term antiseizure medication, most patients were treated by addressing the root metabolic cause for example, pacemaker placement for arrhythmia, lactulose and rifaximin for hyperammonemia, or diazoxide/dietary modification for hypoglycemia. When needed, short-term AEDs such as levetiracetam were introduced in acute cases [40]. Outcomes were generally favorable once the underlying disorder was corrected, and persistent epilepsy did not typically develop.

Overall, this subgroup demonstrates that bariatric surgery can unmask or precipitate seizures secondary to systemic complications, with onset ranging from the immediate postoperative period to a decade later. Careful long-term follow-up is therefore required, particularly for women of reproductive age undergoing sleeve gastrectomy or RYGB.

### 4.6 Limitations

Several limitations should be considered when interpreting the results of this study. Firstly, the quality of the included studies ranged between moderate to good. Secondly, individual studies did not routinely assess metabolic, neurologic factors that might influence effects on postoperative seizure occurrence. Also with the older studies included in this review we need to take into account that there might be a few procedures done in open fashion, despite in current bariatric surgical practice the most procedures are done laparoscopically.

### 4.7 Conclusion

Seizure outcomes after bariatric surgery are variable and influenced by altered drug absorption and postoperative metabolic complications. Most patients maintain seizure control, but a subset develops breakthrough or new-onset seizures, highlighting the need for AEDmonitoring and careful evaluation of metabolic causes.

### 4.8 Take-away messages

- New onset postoperative seizures can occur after both Roux en Y Gastric Bypass, but also Sleeve Gastrectomy
- Postoperatively, special focus should be on preventing metabolic disturbances metabolic disturbances especially hypoglycemia, hyperammonemia, electrolyte abnormalities
- Anti-seizure medication adjustments are more often required in patients after a Roux en Y Gastric Bypass, due to pharmacokinetic changes.

## Consent to participate

Informed consent is not applicable.

## Author contributions

Initial Idea: **MB, SP**.

Literature Search and Data Analysis: **MB, MA, SS, RB, SP, SJSA, CPK**.

Writing the paper: **MB, MA, SS, RB, SP, SJSA, CPK**.

Final Approval: **MB, MA, SS, RB, SP, SJSA, CPK**.

## Ethical review section

This study was a systematic review of previously published literature and did not require Institutional Review Board approval or informed consent.

## Declaration of user of artificial intelligence

In preparing this manuscript, the author utilized AI tools to assist with sentence concision and grammatical review. All content was subsequently reviewed and revised by the authors, who assume full responsibility for the final version of the publication.

## Source of funding

None.

## Conflict of interest

The authors report no conflicts of interest.
